# Profil évolutif de l'insuffisance rénale aiguë chez les personnes vivant avec le VIH à Abidjan, Côte d'Ivoire du 1^er^ janvier 2014 au 31 décembre 2017

**DOI:** 10.11604/pamj.2019.34.2.20016

**Published:** 2019-09-02

**Authors:** Monlet Cyr Guei, Marthe Sidibé, Anastasie Wognin, Serge Didier Konan, Motochi Carole Choho, Hubert Kouamé Yao, Clément Ackoundou-N'guessan, Appolinaire Daze Gnionsahé

**Affiliations:** 1 Service de Néphrologie, CHU de Yopougon, BP 632, Abidjan 21, Côte d'Ivoire; 2 Service de Néphrologie, CHU de Treichville, Kilomètre 1, Boulevard de Marseille, BP V 206, Abidjan, Côte d'Ivoire

**Keywords:** Evolution de la maladie, insuffisance rénale aiguë, infection à VIH, Côte d´Ivoire, Disease outcome, acute renal failure, HIV infection, Ivory Coast

## Abstract

En Côte d'Ivoire, la prévalence de l'infection au virus de l'immunodéficience humaine (VIH) est élevée. Tous les organes peuvent être atteints, en particulier le rein. Certaines études en Côte d'Ivoire ont retrouvé les infections opportunistes comme facteur de mauvais pronostic de l'insuffisance rénale aiguë (IRA). L'objectif de cette étude était de rechercher les éventuels autres facteurs associés à l'évolution de l'IRA chez les personnes infectées par le VIH. Une étude rétrospective, analytique, menée au Centre Hospitalier et Universitaire de Yopougon de janvier 2014 à décembre 2017. Etaient inclus tous les patients âgés de plus de 18 ans, infectés par le VIH et ayant présenté une IRA. Soixante-treize (73) patients ont été inclus, soit une prévalence de 24%. L'âge moyen des patients était de 39,32 ± 10,50 ans avec des extrêmes de 18 et 65 ans. Un sexe ratio de 1,6 en faveur des femmes. L'obésité (p=0,047; OR=8,72; IC (95%)=1,07-39,21) et le taux de CD4< 200/mm^3^ (p=0,000; OR=58,50; IC (95%)=10,31-55,12) étaient associés à un mauvais pronostic de l'IRA. En Côte d'Ivoire, la prévalence hospitalière l'IRA au cours du VIH demeure élevée. Son évolution est défavorable avec le décès ou le passage à la chronicité. En plus de l'immunodépression profonde déjà connue comme facteur de mauvais pronostic, cette étude a montré que l'obésité était associée à une évolution défavorable de l'IRA chez les personnes vivant avec le VIH.

## Abstract (en)

In Ivory Coast, the prevalence of human immunodeficiency virus (HIV) infection is high. Any organ can be affected, in particular the kidney. Some studies conducted in Ivory Coast highlighted that opportunistic infections are a poor prognostic factors for acute renal failure (ARF). The purpose of this study was to investigate the other possible factors associated with ARF outcome among subjects with HIV infection. We conducted a retrospective analytical study, at the University Hospital Center of Yopougon over the period January 2014-December 2017. All patients over the age of 18 years with HIV infection associated with ARF were enrolled. The study included 73 patients, reflecting a rate of 24%. The average age of patients was 39.32 ± 10.50 years, ranging from 18 to 65 years. Sex ratio of 1.6 favoured women. Obesity (p=0.047; OR=8.72; CI (95%)=1.07-39.21) and CD4 count < 200/mm^3^ (p=0.000; OR=58.50; IC (95%)=10.31-55.12) were associated with poor prognosis of ARF. In Ivory Coast, the hospital prevalence of ARF in patients with HIV is high. Unfavorable outcome included death or chronicity. In addition to deep immunosuppression already known as a poor prognostic factor, this study showed that obesity is associated with unfavorable outcome of ARF in subjects with HIV.

## Introduction

L'infection par le virus de l'immunodéficience humaine (VIH) est une affection globale [1]. Dans le monde en 2017, l'ONUSIDA estimait à 36,9 millions le nombre de personnes vivant avec le VIH (pv/VIH) [2]. L'Afrique subsaharienne représente un important foyer endémique de l'infection par le VIH [1]. En côte d'Ivoire, on estimait à 24 000 le nombre de décès dus au VIH-SIDA. La prévalence nationale reste encore élevée 2,7% [3]. Au stade de maladie clinique, tous les organes sont infectés par le virus entrainant ainsi de nombreuses manifestations parmi lesquelles l'atteinte de la fonction rénale [4]. Cette atteinte touche environ 10% des malades infectés et peut survenir à tous les stades de l'infection [5]. Des études rapportent une prévalence entre 2% [6] et 69% [7] de l'insuffisance rénale aiguë (IRA) au cours de l'infection à VIH. Des facteurs comme l'infection par le VIH de type1, le taux de lymphocytes CD4 < 200 éléments/ mm^3^, l'âge avancé, le sexe féminin, le stade III/IV de l'OMS seraient associés à l'IRA [6,8,9]. L'évolution peut souvent être défavorable marquée par une insuffisance rénale terminale (IRT) ou la survenue du décès [6]. Les facteurs associés à cette évolution défavorable de l'IRA sont mal connus dans notre contexte. Par ailleurs, certaines études réalisées en Côte d'Ivoire sur l'association VIH-IRA ont montré que parmi ces facteurs de mauvais pronostic figurent les affections opportunistes liées à une immunodépression profonde [9,10]. Notre étude qui se veut complémentaire, a pour objectif d'étudier les aspects évolutifs et les facteurs associés à l'IRA chez les pvVIH à Abidjan.

## Méthodes

Il s'est agi d'une étude rétrospective à visée analytique, menée dans le service de néphrologie du Centre Hospitalier et Universitaire (CHU) de Yopougon du 1er janvier 2014 au 31 décembre 2017, soit 48 mois. Etaient inclus tous les patients âgés de plus de 18ans, infectés par le VIH et ayant présenté une IRA pendant la période d'étude. N'ont pas été inclus, les patients perdus de vue et ceux ayant un dossier clinique non exploitable. Les données ont été recueillies à l'aide d'une fiche d'enquête standardisée à partir des dossiers médicaux des patients Les paramètres étudiés les données socio-démographiques, cliniques (la pression artérielle, l'indice masse corporelle (IMC), l'état d'hydratation, la protéinurie et l'hématurie à la bandelette, la diurèse et les comorbidités: diabète sucré, hypertension), paracliniques (le taux d'hémoglobine, la créatinine sanguine, l'urée sanguine, le type de VIH, le taux des lymphocytes CD4 et la charge virale), thérapeutiques et évolutives (administration de médicaments néphrotoxiques: AINS, aminosides, produits de contraste iodés, chimiothérapie et/ou médicaments traditionnels) et l'observance du traitement antirétroviral. Un dosage régulier de la créatinine sanguine, au bilan initial, à l'admission, à 3 mois et pendant la période d'étude, a permis d'évaluer l'évolution de l'IRA. L'analyse a été faite à l'aide des logiciels Excel, SPSS (Statistical Package for the Social Sciences) version 22.0 et EPI-INFO dans sa version 3.5. Pour la comparaison des variables qualitatives, nous avons utilisé les tests de Chi carré et exact de Fischer. Le test t de Student a servi à la comparaison des variables quantitatives. Un seuil de signification de p < 0,05 a été retenu.

**Définitions opérationnelles des cas**: l'IRA a été définie par une créatininémie > 15mg/l depuis moins de trois mois et classée selon les critères KDIGO [11]

**IRA stade 1**: créatinine plasmatique ≥26,5 μmol/l ou 1,5 à 1,9 fois la créatinine plasmatique de base ou diurèse < 0,5 ml/kg/h pendant 6 h à 12h.

**IRA stade 2**: créatinine plasmatique 2,0 à 2,9 fois la créatinine plasmatique de base ou diurèse < 0,5 ml/kg/h pendant ≥ 12h.

**IRA stade 3**: créatinine plasmatique 3,0 fois la créatinine plasmatique de base ou créatinine plasmatique ≥354 μmol/l ou mise en route de l'épuration extra-rénale ou diurèse < 0,3ml/kg/h pendant ≥ 24h ou anurie pendant ≥12h.

**L'IRA a été dite fonctionnelle** en présence d'un facteur d'hypoperfusion rénale (diarrhée, vomissements, bas débit cardiaque) ou devant des signes de déshydratation extracellulaire et la protéinurie négative à la bandelette urinaire.

**L'IRA obstructive** devant une IRA associée à une dilatation bilatérale des cavités pyélocalicielles à l'échographie. **L'HTA** est définie par des chiffres tensionnels ≥140mmHg pour la systolique et/ou ≥ 90mmHg pour la diastolique ou chez une personne sous traitement anti hypertenseur avec pression artérielle normale [12]. **L'évolution a été dite favorable** si la créatinine était inférieure à 133 μmol/l (15mg/l) ou si elle diminuait de plus de 50%.

## Résultats

**Figure 1:**
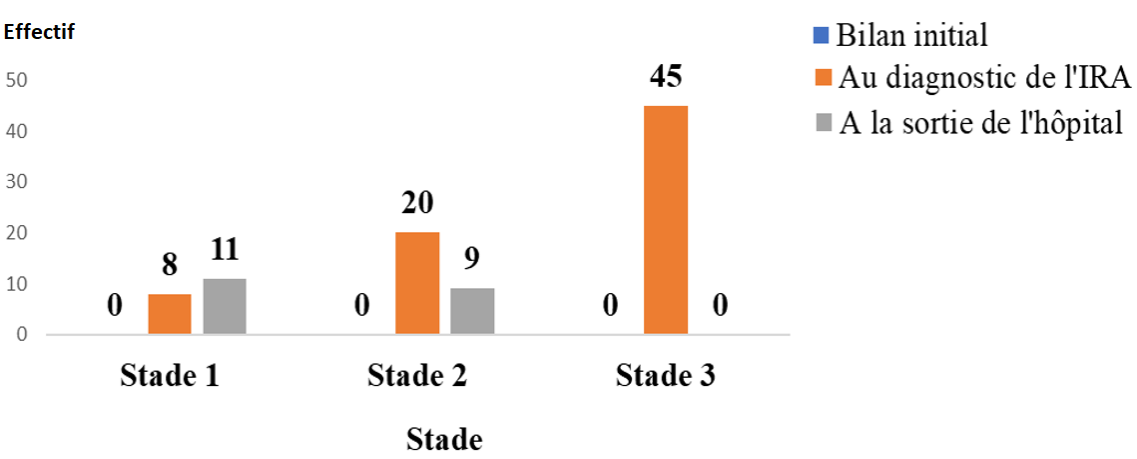
Répartition des patients en fonction du stade d'atteinte rénale selon les KDIGO

**Figure 2:**
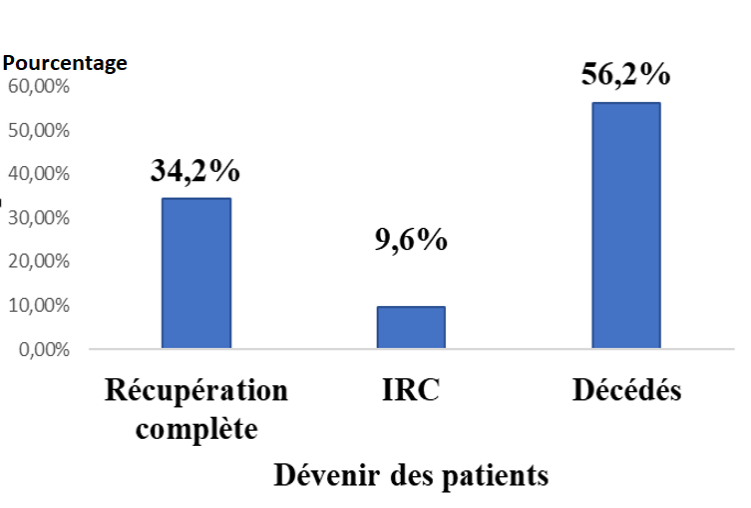
Devenir des patients

**Les caractéristiques générales, cliniques, paracliniques, thérapeutiques et évolutives**: deux mille neuf cent trente-cinq patients ont été hospitalisés dans le service pendant la période d'étude. Parmi eux, 317 patients soit 10,80% étaient infectés par le VIH dont 76(23,9%) avaient présenté une IRA. Notre étude a concerné 73 patients; 3 patients n'ayant pas été inclus, dont 2 perdus de vue et un dossier non exploitable. L'âge moyen de nos patients était de 39,32 ± 10,50 ans avec des extrêmes de 18 et 65 ans. Le sexe féminin était majoritaire avec 62% (Tableau 1, Figure 1, Figure 2).

**Tableau 1:** Les données cliniques, paracliniques, thérapeutiques et évolutives

Paramètres	Effectif (n=73)	Pourcentage (%)
Antécédents médicaux*		
Infections	69	94,5
Automédication	69	94,5
HTA	21	28,8
Diabète	4	5,8
Hépatite virale B	2	2,7
**Indice Masse Corporel**		
Maigre (IMC ≤ 18,5)	36	49,3
Poids normal (IMC: 18,6-24,9)	28	38,3
Surpoids (IMC: 25-29,9)	8	11
Obèse (IMC ≥ 30)	1	1,4
Signes cliniques*		
Déshydratation	66	90,4
Maigreur	36	49,3
HTA	21	28,8
Œdèmes	3	4,1
**Diurèse moyenne journalière (ml)**		
≤ 300	6	8,2
300-500	49	67,2
≥ 500	18	24,6
**Type d’IRA**		
Organique	60	82,2
Fonctionnelle	10	13,7
Obstructive	3	4,1
**Type de VIH**		
VIH_1_	71	97,2
VIH_2_	1	1,4
VIH_1/2_	1	1,4
**Taux de CD4/mm^3^**		
< 200	56	77
200-500	9	12
> 500	8	11
**Traitement par ARV**		
**OUI**	60	82,2
Avec fumarate de ténofovir	45	75
Sans fumarate de ténofovir	15	25
Durée du traitement (mois)		
< 12	41	68,3
13-24	10	16,7
≥ 25	4	6,7
Indéterminée	5	8,3
**NON**	13	17,8
Autres traitements associés*		
Traditionnel	73	100
Cotrimoxazole	31	42,5
Produit de contraste iodé (PCI)	17	23,3
Aminoside	12	16,4
Autres (amoxicilline, métronidazole)	53	72,5
**Devenir des patients**		
Décédés	41	56,2
Vivants	32	43,8

* Plusieurs patients présentaient à la fois plusieurs antécédents médicaux, plusieurs signes cliniques et plusieurs traitements associés

**Les facteurs associés à l'évolution des patients et de la fonction rénale sont répertoriés dans le Tableau 2**.

**Tableau 2:** Récapitulatif des facteurs associés à l’évolution de la fonction rénale

Evolution					
	Vivants (n=32)	Décédés (n=41)	p-value	OR	IC (95 %)
**Indice de masse corporelle (IMC)**					
Maigreur	10(31,2%)	8(19,5%)	0,378	0,53	0,16-1,79
Poids normal	21(65,6%)	23(56,0%)	0,558	0,67	0,23-1,92
Surpoids	1(3,1%)	9(21,9%)	**0,047**	8,72	1,07-39,21
Obésité	0(0%)	1(2,4%)	0,900	0,74	0,01-62,78
**Protéinurie à la bandelette urinaire**					
**Positive**	4(12,5%)	11(26,8%)	0,225	2,57	0,65-12,21
**Hématurie à la bandelette urinaire**					
**Positive**	4(12,5%)	10(24,3%)	0,326	2,26	0,56-10,88
**Antécédent diabète**					
Oui	1(3,1%)	3(7,3%)	0,792	2,45	0,18-132,55
**Antécédent HTA**					
Oui	9(28,1%)	12(29,2%)	0,878	1,06	0,34-3,38
**Hépatite virale B**					
Oui	1(3,1%)	1(2,4%)	0,586	0,77	0,01-62,78
**Automédication moderne**					
Oui	29(90,6%)	40(97,5%)	0,439	4,14	0,31-222,54
**Coinfection HBV**					
Oui	1(3,12%)	1(2,43%)	0,586	0,77	0,01-62,78
**Autres Evènements infectieux**					
Oui	31(96,8%)	3(7,3%)	0,792	0,41	0,01-5,44
**Taux de CD4**					
<200/mm^3^	8(25%)	39(95,1%)	**0,000**	58,50	10,31-55,12
**Régime HAART**					
TDF+3TC+EFV	21(65,6%)	33(80,4%)	0,243	2,16	0,66-7,25
TDF+3TC+NVP	2(6,25%)	1(2,4%)	0,826	0,38	0,01-7,62
TDF+FTC+RTV+AZT	1(3,2%)	0(0%)	0,900	0,00	0,00-30,44

## Discussion

**Caractéristiques sociodémographiques**: l'IRA chez les pvVIH représentait 24 %. Au Sénégal, Cissé *et al.*[13] ont rapporté une prévalence de 12,9% d'IRA chez les pvVIH. Ce taux de prévalence élevé dans notre étude pourrait s'expliquer par le fait que le service de néphrologie du CHU de Yopougon soit situé dans la plus grande commune d'Abidjan et le seul centre de référence des pvVIH souffrant de pathologies rénales dans cette commune. L'âge moyen de nos patients était de 39,32 ± 10,5 ans avec des extrêmes allant de 18 à 65 ans. Cela pourrait s'expliquer par la relative jeunesse de nos populations. Le sexe ratio était de 1,6 dans notre étude. Cette prédominance féminine chez nos patients est corroborée par plusieurs études [1,6,11]. Dans notre étude, ni l'âge, ni le sexe n'était un facteur pronostique de l'IRA.

### Cliniques

**Antécédents**: cette étude a mis en évidence des antécédents pathologiques classiques d'atteinte rénale chez les pvVIH. Ils étaient dominés par les évènements infectieux et l'automédication (95,5%), l'HTA (28,8%), le diabète (5,8%) et l'infection par le VHB (2,7%). Mais ils n'étaient pas associés à l'évolution défavorable de l'IRA. Leur existence chez les pvVIH, interpelle encore plus les praticiens à aller au-delà de la spécificité du risque rénal liée au VIH et son traitement.

**Profil sérologique**: la majorité de nos patients avait une infection à VIH1 soit 97,2%. Ce résultat corrobore à celui des données sur la prévalence mondiale du VIH1 [1]. Cependant le type de VIH n'est pas un facteur de risque pour les complications rénales [14]. Dans notre étude le type de VIH n'est pas un facteur associé à l'évolution péjorative de l'insuffisance rénale aiguë.

**L'indice de masse corporelle (IMC)**: environ un patient sur deux était amaigri. Ce constat témoigne de l'altération de l'état général en rapport avec le stade d'évolution de l'infection VIH. Dans notre étude le surpoids était un facteur défavorable de l'IRA (P=0,047).

### Paraclinique

**Description de la fonction rénale**: à l'initiation du traitement ARV, aucun patient ne présentait une IRA. Au diagnostic de l'IRA: 45 patients (61,6%) présentaient une IRA au Stade 3 de la classification des KDIGO; 20 Patients (27,37%) présentaient IRA au stade 2 et 8 patients (10,95%) présentaient une IRA au stade 1. A la sortie de l'hôpital, aucun patient n'était au stade 3; 9 patients (12,32%) présentaient IRA stade 2 et 11 patients (34,37%) au stade 1.

**Taux de CD4**: l'insuffisance rénale peut survenir à tous les stades de l'infection par le VIH, notamment en cas d'immunodépression profonde [5]. 77% des patients avait un taux de CD4 <200 cellules/mm3 et l'évolution de l'insuffisance rénale était défavorable quel que soit le taux de CD4 (P= 0,001).

**Traitement**: les études réalisées aux Etats-Unis et en France ont rapportés le risque rénal du traitement par les ARV principalement par le ténofovir (TDF), dont l'impact sur le rein serait au même niveau sinon supérieur aux effets rénaux d'une association d'antirétroviraux. Quatre-vingt-deux virgule deux pourcent de nos patients étaient sous ARV, parmi les schémas thérapeutiques, 75% d'entre eux contenaient du TDF qui nécessitait l'ajustement de doses; également le cotrimoxazole, les aminosides et d'autres traitement (métronidazole, amoxicilline) respectivement 42,5%, 16,4% et 72,5%.

**Cas particulier du TDF**: parmi les antirétroviraux pouvant potentiellement augmenter le risque d'atteinte rénale, le cas du TDF est à souligner. Bien que l'incidence des effets rénaux soit très faible dans les essais cliniques [15], de nombreux cas de toxicité rénale ont été rapportés du fait de sa large utilisation. Chez les patients traités par du TDF, le risque de protéinurie, de diminution du DFG et également d'insuffisance rénale chronique est augmenté [16]. Cependant dans notre étude, l'utilisation du TDF dans les différents schémas thérapeutiques n'était statistiquement pas associée à une évolution défavorable de l'IRA.

**Devenir des patients et évolution de la fonction rénale**: dans notre étude, 43,8% des patients étaient vivants. Le taux de mortalité a été de 56,2%. Dans la littérature, la mortalité hospitalière chez les pvVIH varie entre 27% [17] et 46,7% [14]. La mortalité dans notre étude est supérieure à celle des pays développés qui est de 14% [18]. Ces chiffres s'expliqueraient par retard de la consultation, l'insuffisance des plateaux techniques et de la pénurie de personnels de santé spécialisés [19]. L'évolution de l'IRA s'est faite vers la récupération de la fonction rénale chez 34,2%. Le passage vers la chronicité a été noté chez 9,6% des patients et le taux de décès était de 56,2%. Une étude a retrouvé comme facteur de risque d'IRC chez les pvVIH le sexe féminin, l'âge, un diabète, une hyperlipidémie, un taux bas de lymphocytes CD4 < 200/mm^3^ et l'exposition au ténofovir supplémentaire [20].

**Traitements associés**: la totalité des patients avaient bénéficié de la tradithérapie. Une grande partie des patients soit 72,5% avaient associées autres médicaments à leur traitement ARV tels Amoxicilline, Métronidazole. Cette association pourrait se justifier par l'apparition d'infections opportunistes liées à l'immunodépression par le VIH. 23,3% des patients ont utilisés des PCI au cours du traitement ARV. L´IRA secondaire aux PCI fait partie des trois principales causes d'IRA acquise à l'hôpital [14]. La fréquence d'IRA secondaires aux PCI dans la population générale varie entre un et deux pourcents [21]. Le rein est un organe impliqué dans l'élimination des médicaments. Toute défaillance de celui-ci peut contre-indiquer l'utilisation temporaire ou définitive d'un médicament néphrotoxique ou nécessiter la mise en place de mesures spécifiques (prévention de la toxicité rénale, adaptation de la posologie) [14].

**Facteurs associés classiques d'atteinte rénale**: l'IMC calculé chez les patients a mis en évidence une obésité dans 1,4% et un surpoids dans 11%. La corrélation entre l'IMC et la survenue de l'IRA était significative. L'excès de poids constitue un risque de maladie rénale. L'obésité et le surpoids sont des facteurs de risque indépendants, Bien que la prévalence des maladies rénales en relation avec l'obésité ne soit pas clairement définie, des études ont montré une corrélation significative entre l'IMC d'une part et la survenue d'une protéinurie [22] et/ou d'une insuffisance rénale d'autre part [23]. Au plan thérapeutique, l´étude de Chagnac *et al.* [24] a clairement montré l'effet bénéfique de la perte de poids sur la fonction rénale et la protéinurie. La majorité de nos patients (77%) avait un taux de CD4<200/mm^3^. Le taux de CD4 bas est associé à l'apparition d'infections opportunistes liées à une immunodépression profonde [9,10] chez le pvVIH présentant une IRA.

## Conclusion

La prévalence hospitalière l'IRA au cours du VIH demeure élevée, estimée dans notre étude à 24%. Nous avons noté une prédominance féminine à 62%. L'évolution de l'IRA est défavorable avec la survenue de décès ou le passage au stade de chronicité. Les facteurs associés à cette évolution défavorable de l'IRA sont le surpoids et l'immunodépression par le VIH avec un taux de CD4<200.

### Etat des connaissances actuelles sur le sujet

- Le VIH de type1, le taux de lymphocytes CD4 < 200 éléments/ mm^3^, l'âge avancé, le sexe féminin sont associés à une évolution défavorable de l'IRA;
- Les stades III/IV de l'OMS de l'infection à VIH sont associés à un mauvais pronostic de l'IRA;
- En Côte d'Ivoire sur l'association VIH-IRA, il a été montré que les affections opportunistes sont un facteur de mauvais pronostic de l'IRA.

### Contribution de notre étude à la connaissance

- Le VIH de type1, le taux de lymphocytes CD4 < 200 éléments/ mm3, l'âge avancé, le sexe féminin sont associés à une évolution défavorable de l'IRA;
- Les stades III/IV de l'OMS de l'infection à VIH sont associés à un mauvais pronostic de l'IRA;
- En Côte d'Ivoire sur l'association VIH-IRA, il a été montré que les affections opportunistes sont un facteur de mauvais pronostic de l'IRA.

## Conflits des intérêts

Les auteurs ne déclarent aucun conflit d’intérêts.

## References

[1] Canada. Agence de la santé publique Actualités en épidémiologie du VIH/sida: le VIH/sida chez les personnes originaires de pays où le VIH est endémique.

[2] ONUSIDA Fiche d'information-journée mondiale du sida 2018, statistiques mondiales sur le vih en 2017.

[3] ONUSIDA Côte d'Ivoire/ONUSIDA.

[4] Nyimi ML, Lepira FB, Sumaili KE, Ebengo BC, Nseka MN, Longo-Mbenza B (2001). Insuffisance rénale aiguë associée à l'infection par le VIH à Kinshasa: à propos de 24 observations. Louvain Méd.

[5] Ekat HM, Courpotin C, Diafouka M, Akolbout M, Mahambou-Nsonde D, Bitsindou PR (2013). Prévalence et facteurs associés à l'insuffisance rénale chez les patients nouvellement dépistés VIH-positifs à Brazzaville (République du Congo). Med et Sante Trop.

[6] Yao KH, Sanogo S, Doumbia A, Konan S-D, N'zoué KS, Diallo D (2018). Acute kidney injury in hospitalized HIV-infected patients living in Cote d'Ivoire. J Renal Endocrinol.

[7] Kaba ML, Condé A, Soumah M, Cissé M, Traoré M (2016). Prévalence des insuffisances rénales aiguë et chronique chez les patients infectés par le VIH à Conakry. Néphrol Ther.

[8] Kaba ML, Onna EE, Cissé M, Tounkara T, Bah A, Traoré M (2013). Débit de filtration glomérulaire calculé par MDRD chez les patients infectés par le VIH à l'induction du traitement antirétroviral à Conakry. Néphrol and Therapeutique.

[9] Tourret J, Tostivint I, Deray G, Isnard-Bagnis C (2009). Néphropathies rencontrées au cours de l'infection par le virus de l'immunodéficience humaine (VIH). Néphrol Ther.

[10] Yao KH, Tanon AK, Lagou AD, Konan SD, Diopoh SP, Meite F (2017). Étude comparative de l'insuffisance rénale aiguë communautaire chez le sujet VIH positif et le sujet VIH négatif: expérience d'un service de médecine interne à Abidjan (Côte d'Ivoire). Néphrol Ther.

[11] Machado MN, Nakazone MA, Maia LN (2014). Acute kidney injury based on KDIGO (Kidney Disease Improving Global Outcomes) criteria in patients with elevated baseline serum creatinine undergoing cardiac surgery. Rev Bras Cir Cardiovasc.

[12] Aram V, Chobanian MD (2017). Guidelines for the Management of Hypertension. Med Clin N Am.

[13] Cisse M, Fall K, Ka EHF, Lemrabott AT, Faye M, Karim DA (2015). Atteintes rénales au cours de l'infection à VIH à Dakar: à propos de 32 cas. Néphrol Ther.

[14] Conseil national du sida et des hépatites virales et l'Agence nationale de recherche sur le sida et les hépatites virales (2016). France: recommandations du groupe d'experts pour la prise en charge des personnes vivant avec le VIH.

[15] Ondounda M, Tanon A, Ehui E, Ouattara I, Kassi A, Aba YT (2011). Le syndrome de Fanconi induit par le ténofovir en Afrique: deux cas en Côte d'Ivoire. Med Mal Infect.

[16] Scherzer R, Estrella M, Li Y, Choi AI, Deeks SG, Grunfeld C (2012). Association of tenofovir exposure with kidney disease risk in HIV infection. AIDS.

[17] Wyatt CM, Arons RR, Klotman PE, Klotman ME (2006). Acute renal failure in hospitalized patients with HIV: risk factors and impact on in-hospital mortality. AIDS.

[18] Flexor Gabriella, Zucman David, Berthé Huguette, Meier Françoise, Force Gilles, Greder-Belan Alix (2013). Vieillissement et infection par le VIH: suivi de 149 patients âgés de plus de 60 ans infectés par le VIH (COREVIH* Île-de-France Ouest). Presse Med.

[19] Ly A (2011). Enjeux et perspectives de la prévention des cancers dans les pays en développement. J Afr Cancer.

[20] Deti KE, Vandenhende MA, Dauchy F, Michaux C, Geffard S, Lazaro E (2009). Incidence et facteurs de risque de l'insuffisance rénale chronique chez les patients infectés par le VIH: étude de cohorte de 2613 patients (2004-2008). Rev Med Interne.

[21] Pironi Bruno, Roccia Maria Grazia, Bianchi Maria, Jahaira Carolina Aracena, Fioranelli Massimo (2015). Contrast-induced nephropathy. J Integr Cardiol.

[22] Cohen Arthur H (1999). Pathology of Renal Complications in Obesity. Curr Hypertens Rep.

[23] Geneidy A, Solomon R (2006). Obesity and Renal Disease. Obesity and Diabetes.

[24] Mori H, Okada Y, Arao T, Tanaka Y (2014). Sitagliptin improves albuminuria in patients with type 2 diabetes mellitus. J Diabetes Investig.

